# Animal Experimental Models in Bone Metabolic Disease

**DOI:** 10.3390/ijms24119534

**Published:** 2023-05-31

**Authors:** Ricardo Usategui-Martín, José Luis Pérez-Castrillón

**Affiliations:** 1 Department of Cell Biology, Genetics, Histology, and Pharmacology, Faculty of Medicine, University of Valladolid, 47003 Valladolid, Spain; ricardo.usategui@uva.es; 2 IOBA, University of Valladolid, 47011 Valladolid, Spain; 3 Service of Internal Medicine, Río Hortega University Hospital, 47012 Valladolid, Spain; 4 Department of Medicine, Dermatology and Toxicology, Faculty of Medicine, University of Valladolid, 47003 Valladolid, Spain

Bone is a highly specialized and dynamic tissue with several crucial functions, including support, movement support, protection of vital organs, and mineral storage. Continuous remodeling allows the bone to function healthily and maintains bone strength [1]. The bone remodeling cycle is 120 days and involves the removal of mineralized bone by osteoclasts, followed by the formation of a new bone matrix by osteoblasts. Systemic and local factors regulate bone remodeling, and the phases of bone remodeling are the initiation–activation of site-specific remodeling, the resorption phase (the osteoclasts remove the mineralized bone), the reversal phase (mesenchymal stem cells and osteoblasts progenitors are recruited on the bone surface), the formation phase (osteoblasts are activated, and a new bone matrix is synthesized), and mineralization [2,3].

Bone metabolic diseases are a broad spectrum of clinically different diseases that share the common finding of an aberrant bone metabolism leading to a defective skeleton and bone abnormalities. Osteoporosis is the most common bone metabolic disease worldwide. Osteoporosis is characterized by low bone mineral density (BMD), reduced bone mass, and the alteration of bone microarchitecture, leading to bone fragility and an increased risk of fracture. It is a silent, progressive systemic metabolic disease with important clinical, social, and economic consequences. Osteoporosis is associated with a worsened quality of life and increased disability, morbidity, and mortality [4,5].

It is important to understand the molecular mechanisms involved in the appearance of bone metabolic diseases to allow for the establishment of therapeutic targets. Expanded knowledge of its pathophysiology leads to a better understanding of the disease and its solutions. Knowledge of these new therapeutic targets will allow for the design of new therapeutic options. These must be tested in experimental models before being tested in humans. In addition, the therapeutic options approved for other diseases could be beneficial in metabolic bone disease, and tests on animal models would facilitate their use.

The use of animal models in studying human anatomy and physiology dates back to the 6th century BCE, and their use in the pursuit of biomedical knowledge has continued for millennia [6]. Animal models are essential to advance the knowledge of the pathophysiological mechanisms of diseases and in the establishment of new therapeutic strategies. The remarkable anatomical and physiological similarities between humans and animals, particularly mammals, have prompted researchers to investigate numerous mechanisms and assess novel therapies in animal models before applying them to humans [7,8]. In addition, the relationship between animal models and human studies is reciprocal; both provide important information about biology and behavior, guide the direction of the other in research, and complement findings about one another [7,8].

Animal models of bone disorders are crucial to advancing the knowledge of the underlying pathophysiology of these diseases, and testing new treatment strategies. Despite the advances in bone metabolism using in vitro models, the use of animals is crucial because in vitro studies cannot fully reproduce the physiological behavior, bone physiology, and pathology of the organisms. The vast majority of these models are focused on the study of bone loss. Several species are used in these studies, ranging from small laboratory rodents to non-human primates. Mice and rats are probably the most used animals to study bone metabolic diseases. Although several mouse and rat models of bone loss have been developed, the mouse model of post-menopausal osteoporosis from ovariectomy is the most used [9]. Post-menopausal osteoporosis from ovariectomy was also studied in a sheep model [10]. Other mouse or rat models of bone loss are based on skeleton disuse, lactation-induced bone loss, glucocorticoid excess, bone loss associated with erythropoietin receptor signaling, or models of bone loss associated with a hypoxia environment [9,11]. Skeletal alterations associated with hypoxia were also studied in guinea pig models [12]. The principal clinical consequence of bone loss is a fracture; therefore, there are also several animal models for studying bone fracture healing [13]. Osteoarthritis (OA) is a degenerative joint disease that can affect the many tissues of the joint. OA is a painful and chronic pathology characterized by the loss of articular cartilage, sclerosis of the subchondral bone, hyperplasia, and inflammation of the synovium [14]. Animal models for studying OA are also frequently used to better understand the pathophysiological process and treatment development. Small and large animals are used. Small animals are used for studying the pathogenesis of OA due to how they show more rapid disease progression. On the other hand, large animals such as dogs or goats are used to evaluate new OA treatments due to their anatomical similarity to humans [15,16].

Using animals to emulate human pathologies is crucial in biomedical research, as they allow us to study pathophysiology or test new drugs yielding robust results because, for example, they can achieve homogeneity between subjects. However, it is also important to note that animal models also have some limitations. The principal limitation may be that no ideal animal model of human disease exists; no animal can perfectly imitate human anatomy or physiology. It is, therefore, important to know the strengths and limitations of animal models before conducting research with them.
